# Elevated plasma IL-6 and CRP levels are associated with adverse clinical outcomes and death in critically ill SARS-CoV-2 patients: inflammatory response of SARS-CoV-2 patients

**DOI:** 10.1186/s13613-020-00798-x

**Published:** 2021-01-13

**Authors:** Jean-Rémi Lavillegrand, Marc Garnier, Agathe Spaeth, Nathalie Mario, Geoffroy Hariri, Antoine Pilon, Enora Berti, Fabienne Fieux, Sara Thietart, Tomas Urbina, Matthieu Turpin, Lucie Darrivere, Muriel Fartoukh, Franck Verdonk, Guillaume Dumas, Alain Tedgui, Bertrand Guidet, Eric Maury, Yannick Chantran, Guillaume Voiriot, Hafid Ait-Oufella

**Affiliations:** 1 Service de Médecine Intensive-Réanimation, Hôpital Saint-Antoine, Assistance Publique-Hôpitaux de Paris, 184 rue du faubourg Saint-Antoine, 75571 Paris cedex 12, France; 2 Sorbonne Université, Paris, France; 3 Service D’Anesthésie-Réanimation, Hôpital Saint-Antoine, Assistance Publique-Hôpitaux de Paris, Paris, France; 4 Département de Biochimie, Hormonologie et Suivi Thérapeutique, Hôpital Saint-Antoine, Assistance Publique-Hôpitaux de Paris, Paris, France; 5 Service de Médecine Intensive-Réanimation, Hôpital Tenon, Assistance Publique-Hôpitaux de Paris, Paris, France; 6 Inserm U970, Cardiovascular Research Center, Université de Paris, Paris, France; 7 Département D’Immunologie Biologique, Hôpital Saint-Antoine, Assistance Publique-Hôpitaux de Paris, Paris, France

**Keywords:** SARS-CoV-2, Covid-19, Cytokine, Inflammation, Outcome

## Abstract

**Background:**

SARS coronavirus 2 (SARS-CoV-2) is responsible for high morbidity and mortality worldwide, mostly due to the exacerbated inflammatory response observed in critically ill patients. However, little is known about the kinetics of the systemic immune response and its association with survival in SARS-CoV-2+ patients admitted in ICU. We aimed to compare the immuno-inflammatory features according to organ failure severity and in-ICU mortality.

**Methods:**

Six-week multicentre study (*N* = 3) including SARS-CoV-2+ patients admitted in ICU. Analysis of plasma biomarkers at days 0 and 3–4 according to organ failure worsening (increase in SOFA score) and 60-day mortality.

**Results:**

101 patients were included. Patients had severe respiratory diseases with PaO2/FiO2 of 155 [111–251] mmHg), SAPS II of 37 [31–45] and SOFA score of 4 [3–7]. Eighty-three patients (83%) required endotracheal intubation/mechanical ventilation and among them, 64% were treated with prone position. IL-1β was barely detectable. Baseline IL-6 levels positively correlated with organ failure severity. Baseline IL-6 and CRP levels were significantly higher in patients in the worsening group than in the non-worsening group (278 [70–622] vs. 71 [29–153] pg/mL, *P* < 0.01; and 178 [100–295] vs. 100 [37–213] mg/L, *P* < 0.05, respectively). Baseline IL-6 and CRP levels were significantly higher in non-survivors compared to survivors but fibrinogen levels and lymphocyte counts were not different between groups. After adjustment on SOFA score and time from symptom onset to first dosage, IL-6 and CRP remained significantly associated with mortality. IL-6 changes between Day 0 and Day 3–4 were not different according to the outcome. *A contrario*, kinetics of CRP and lymphocyte count were different between survivors and non-survivors.

**Conclusions:**

In SARS-CoV-2+ patients admitted in ICU, a systemic pro-inflammatory signature was associated with clinical worsening and 60-day mortality.

## Background

Coronaviruses are a family of viruses responsible for Middle-East Respiratory Syndrome (MERS) and the Severe Acute Respiratory Syndrome (SARS) outbreaks [1, 2]. SARS coronavirus 2 (SARS-CoV-2) is a member of Coronavirus family, first identified in Wuhan by the Chinese Center for Disease. It is currently responsible for the COVID-19 worldwide pandemic, which has resulted in high rates of hospitalisation and intensive care unit (ICU) admission [3].

Most patients with SARS-CoV-2 infection experience mild to moderate respiratory illnesses with various symptoms such as fatigue, fever, dry cough, shortness of breath and recover without requiring special treatment. However, about 5–10% of patients further develop life-threatening acute respiratory distress syndrome [4]. The pathophysiology of SARS-CoV-2 -induced lung damage is largely uncharted. The direct cytotoxicity of the virus on pulmonary endothelial and alveolar epithelial cells, associated with the aftermath of an intense systemic inflammatory response could cause severe forms. Published studies from China suggest that SARS-CoV-2 strongly modulates the immune response, leading to lymphopenia and life-threatening systemic release of pro-inflammatory cytokines [4, 5]. More recent studies in Europe [6, 7] and America [8] also found that high IL-6 levels at admission are associated with poor outcome in hospitalized patients with SARS-CoV-2. However, cytokine profiles and kinetics of critically ill patients with severe disease admitted in ICU, as well as the relationship between inflammatory biomarkers and disease course in this population, remain poorly investigated.

In this study, we performed an analysis of a predefined set of inflammatory biomarkers on 101 patients with SARS-CoV-2 infection admitted to 3 French Intensive Care Units. We measured plasma levels of anti-inflammatory cytokine interleukin 10 (IL-10), and pro-inflammatory cytokines interleukin 1β (IL-1β), tumor necrosis factor α (TNF-α) and interleukin 6 (IL-6) that could be targeted (directly or through receptor blockade) by commercially available neutralizing monoclonal antibodies. We tested the hypothesis that pro-inflammatory cytokines are associated with both disease severity and outcome in critically ill patients with SARS-CoV-2 infection and that the dynamics of inflammatory makers over the first 3–4 days of ICU management may provide additional information.

## Methods

### Study scheme

Patients with confirmed SARS-CoV-2 infection referred to Saint-Antoine hospital and Tenon Hospital (Paris, France), and subsequently admitted to one of the ICUs (*N* = 3) between 1st March and 15th April 2020 were enrolled. Laboratory confirmation for SARS-CoV-2 was defined as a positive result of real-time reverse transcriptase–polymerase chain reaction (RT-PCR) assay of nasal, pharyngeal, or lower respiratory tract samples. Plasma levels of four cytokines (IL-10, IL-1β, TNF-α and IL-6), C-reactive protein (CRP) and fibrinogen were measured within the first days of ICU admission (Day 0) and 3–4 days later (day 3–4). Cytokines were measured by ELISA (Quantikine ELISA Kit, R&D Systems) on plasma according to the appropriate dilution and following recommendations of the manufacturer. Time between plasma isolation and freezing procedure was less than 2 h. Thresholds detection were 7.8 pg/mL for IL-10, 3.9 pg/mL for IL-1β, 15.6 pg/mL for TNF-α and 3.13 pg/mL for IL-6.

### Data collection

We collected and analysed medical history, physical examination, and haematological, biochemical and immunological data obtained in patients with SARS-CoV-2 infection from electronic medical records. Data collection forms were anonymised and reviewed independently by two investigators. Data collected at ICU admission included age, gender, cardiovascular risk factors, comorbidities (obesity defined by a body mass index > 30 kg.m^−2^, hypertension, diabetes, cardiovascular disease, chronic respiratory disease, chronic kidney disease defined by a creatinine clearance < 60 ml.min^−1^.1.73 m^−2^). Disease severity was evaluated using Simplifier Acute Physiology Score (SAPS II) [9], Sequential Organ Failure Assessment without neurological points (called SOFA^#^) [10] and organ support therapy were measured at Baseline (Day 0). Acute kidney injury was defined according to KDIGO definition (Stage ≥ 2) [11]. Disease severity worsening was defined as an increase of SOFA^#^ score ≥ 1 point between day 0 and day 3–4. Mortality was evaluated at day 60 after ICU admission.

### Statistical analysis

Continuous variables are described as median and interquartile range (IQR) and compared using Wilcoxon’s rank sum test or the Kruskal–Wallis test; categorical variables are summarized by counts (percents) and compared using exact Fisher test or Pearson’s Chi-square test. Spearman correlation coefficient was used to test correlation between baseline cytokines values and others continuous data.

To investigate the relationship between cytokines or inflammatory biomarkers and outcome, we performed the following analyses. First, we studied differences in each cytokine levels (IL-6, IL-1β, TNF-α and IL-10) at baseline (eg Day 0) according to the time from symptoms onset (as a dummy variable, < 9 days, 9–19 days and > 19 days) and organ failure severity at ICU admission. Second, we studied the relationship between baseline biomarkers values and worsening of organ failure at day 3–4 (defined as an increase of SOFA score ≥ 1 point). Third, we investigated the prognosis value of biomarkers on day 60 mortality. For each biomarkers, we computed Hazard ratio (HR) of mortality using Cox model adjusted on SOFA score and the time from respiratory symptoms onset. Receiver operating characteristics curves (ROC) were plotted for day 60 mortality. The accuracy of these variables was assessed calculating its area under the curve (AUC), assessment of the best cut-off value, sensitivity and specificity calculation as well as the likelihood ratios.

To evaluate the relationship between patient status at day 60 and biomarker kinetics, we modeled biomarkers as a function of time, by fitting a linear mixed model, to take into account the clustering aspect of repeated data. These models allowed us to test for differences in average biomarker levels between survivors and non-survivors and investigate trends over time according to vital status. Then, we computed the Delta change for each biomarker (value at day3 minus value at Day 0) and the biomarker ratio (eg the percentage of change per day relative to the first measurement). These variables were used together with SOFA at admission and time from symptom onset in a Cox model for day 60 mortality.

Additionally, to identify variables associated with day-60 mortality, we built univariate Cox regression models. Clinically relevant variables significantly associated with day-60 mortality (*p* < 0.01) were entered in multivariable model.

For each model, collinearity between variables and pairwise interactions were tested. Linearity of continuous variable and proportional Hazards assumption were checked. Multivariate Cox regression selection were performed with stepwise selection based on AIC. Because missing value accounted for less than 10%, analyses were performed on complete cases.

All tests were two-sided, and P values less than 0.05 were considered as statistically significant. Statistics were performed using R (https://www.R-project.org/) software, and graphical representations were performed using GraphPad Prism 5.04 (Graph Pad Software Inc. ®).

## Results

### Patients

Over a 6-week period, 150 patients with SARS-CoV-2 infection were admitted in 3 ICUs. Cytokine profiles were available in 130 patients. Twenty-nine patients receiving compassionate immunomodulating treatments (anti-cytokine neutralizing monoclonal antibodies) before ICU admission were excluded. Among the 101 patients which were ultimately included in our analysis (Additional file 1), 82 were men (82%), with a mean age of 59 ± 11 years. Patient characteristics are summarised in Table 1. At Day 0, patients with SARS-CoV-2 infection admitted in ICU had mainly severe respiratory diseases with PaO2/FiO2 of 155 [111–251] mmHg, SAPS II of 37 [31–45] and modified SOFA score of 4 [3–7]. At day 0, Eighty-three patients (83%) required endotracheal intubation/mechanical ventilation, and among them 53 (64%) were treated with prone positioning during ICU stay. Patient biological characteristics are summarised in Additional file 2.

**Table 1:** Characteristics of included ICU patients with SARS-CoV-2 infection according to 60-day outcome

Characteristics	Total *N* = 101	Survivors *N* = 82	Non-survivors *N* = 19	*P* value
Men (N, %)	82 (82%)	68 (83%)	14 (74%)	0.35
Age (years)	59 ± 11	57 ± 10	63 ± 11	0.07
SOFA^#^ score	4 [3–7]	4 [3–6]	6 [4–8]	0.005
SAPS II	37 [31–45]	37 [31–44]	41 [33–48]	0.45
Body mass index (kg/m^2^)				
< 30	63 (63%)	53 (65%)	10 (52%)	
> 30	38 (38%)	30 (37%)	8 (42%)	0.65
Comorbidity (*N*, %)				
Hypertension	51 (51%)	44 (54%)	7 (36%)	0.18
Diabetes mellitus	29 (29%)	23 (28%)	6 (34%)	0.76
Cardiovascular disease	12 (12%)	10 (12%)	2 (11%)	0.84
Chronic respiratory disease	18 (18%)	15 (18%)	3 (16%)	0.81
Chronic kidney disease	14 (14%)	11 (13%)	3 (16%)	0.78
Cirrhosis	2 (2%)	2 (2%)	0	0.49
Previous cancer	2 (2%)	2 (2%)	0	0.49
Haematological malignancy	6 (6%)	5 (6%)	1 (5%)	0.89
Treatment (*N*, %)				
Glucocorticoids	19 (19%)	16 (19%)	3 (16%)	0.71
ACE inhibitors	28 (28%)	22 (25%)	6 (31%)	0.67
Organ support therapy at day 0 (*N*, %)				
Sedative drugs	71 (71%)	55 (67%)	16 (84%)	0.14
Neuromuscular blockers	57 (57%)	44 (54%)	13 (68%)	0.24
Mechanical ventilation	83 (83%)	67 (82%)	16 (84%)	0.79
Prone positioning	53 (53%)	42 (51%)	9 (47%)	0.76
Haemodialysis	11 (11%)	10 (12%)	1 (5%)	0.38
Norepinephrine				
*N* (%)	33 (33%)	24 (29%)	9 (47%)	0.13
Dosage (mg/h)	0 [0–0.22]	0 [0–0.1]	0.1 [0–0.87]	0.07
Co-infections at day 3 (*N*, %)	37 (37%)	30 (36%)	7 (37%)	0.98

Data are expressed as *N* (%) or median (1st IQR–3rd IQR). *ACE* Angiotensin-Converting Enzyme, *NS* non-significant

### Immuno-inflammatory profile according to time from symptom onset to dosage

**Fig. 1:**
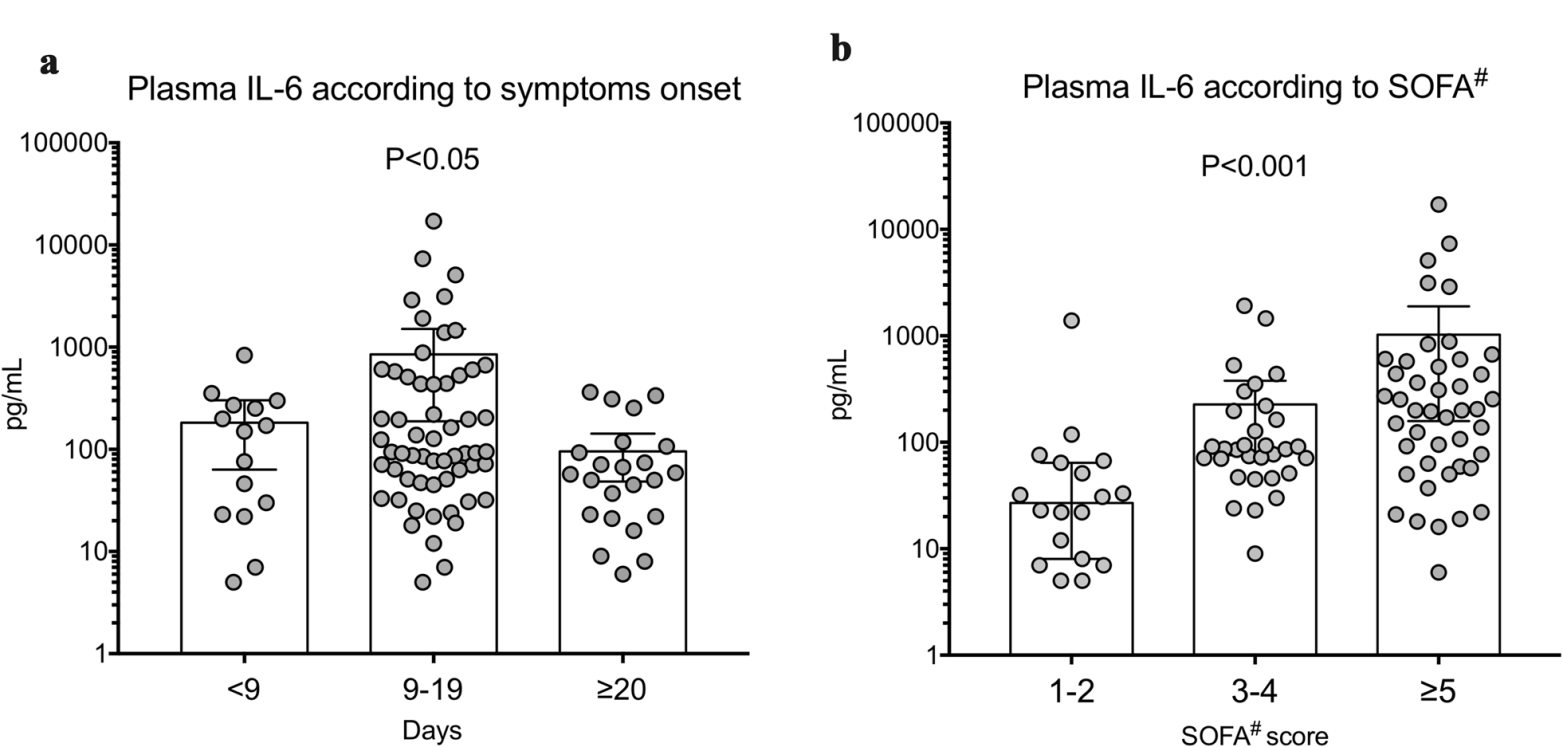
A, Plasma levels of IL-6 according to time between symptom onset and cytokine measurement (Kruskal–Wallis Test). B, Plasma levels of IL-6 according to organ failure severity, evaluated using SOFA^#^ score (SOFA score without neurological points). Data are expressed as median with 95% CI

Cytokine baseline plasma levels were measured 14 [11–20] days after the onset of symptoms. First, we analysed plasma levels of cytokines according to time elapsed from the onset of symptoms and blood sampling. IL-1β was barely detectable (Additional file 3), IL-10 and TNF-α plasma levels were not different according to symptom duration (Additional file 4). IL-6 plasma levels significantly varied according to symptom duration, being significantly higher in patients from whom blood samples were collected between days 9 and 19 after symptom onset (*P* = 0.03) (Fig. 1a).

### Immuno-inflammatory profile according to organ failure severity

**Fig. 2:**
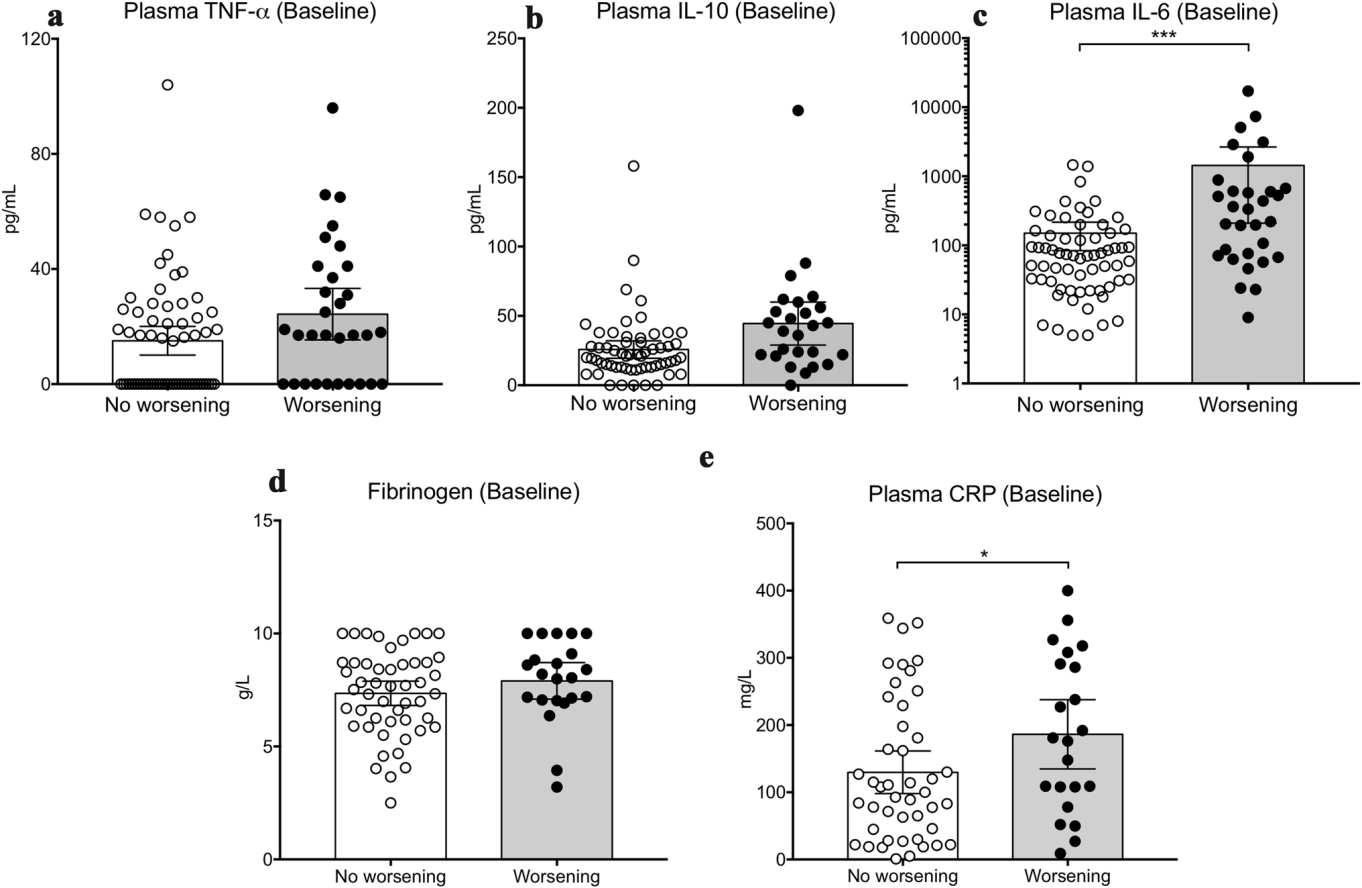
Plasma levels of TNF-α (**a**), IL-10 (**b**), IL-6 (**c**), Fibrinogen (**d**) and CRP (**e**) according to organ failure worsening within the next 3 days. Data are expressed as median with 95% CI. *, *P* < 0.05, ***, *P* < 0.005

Baseline IL-6 plasma levels positively correlated with organ failure severity (Fig. 1b and Additional file 5) but did not correlate with CRP levels (Additional file 6). Next, we analysed the relationship between baseline plasma levels of inflammation markers and organ failure worsening between days 0 and 3–4. Organ failure worsening, defined as an increase of SOFA score ≥ 1 point, was observed in 32 patients (32%) (Additional file 1). Baseline plasma levels of TNF-α (Fig. 2a), IL-10 (Fig. 2b) and fibrinogen (Fig. 2d) were not different between patients in the worsening and non-worsening groups, while IL-6 and CRP plasma levels were significantly higher in the worsening group: 278 [70–622] vs. 71 [29–153] pg/mL, *P* = 0.003 for IL-6 (Fig. 2c) and 178 [100–295] vs. 100 [37–213] mg/L, *P* = 0.04 for CRP (Fig. 2e).

### Immuno-inflammatory profile according to in-ICU survival

**Fig. 3:**
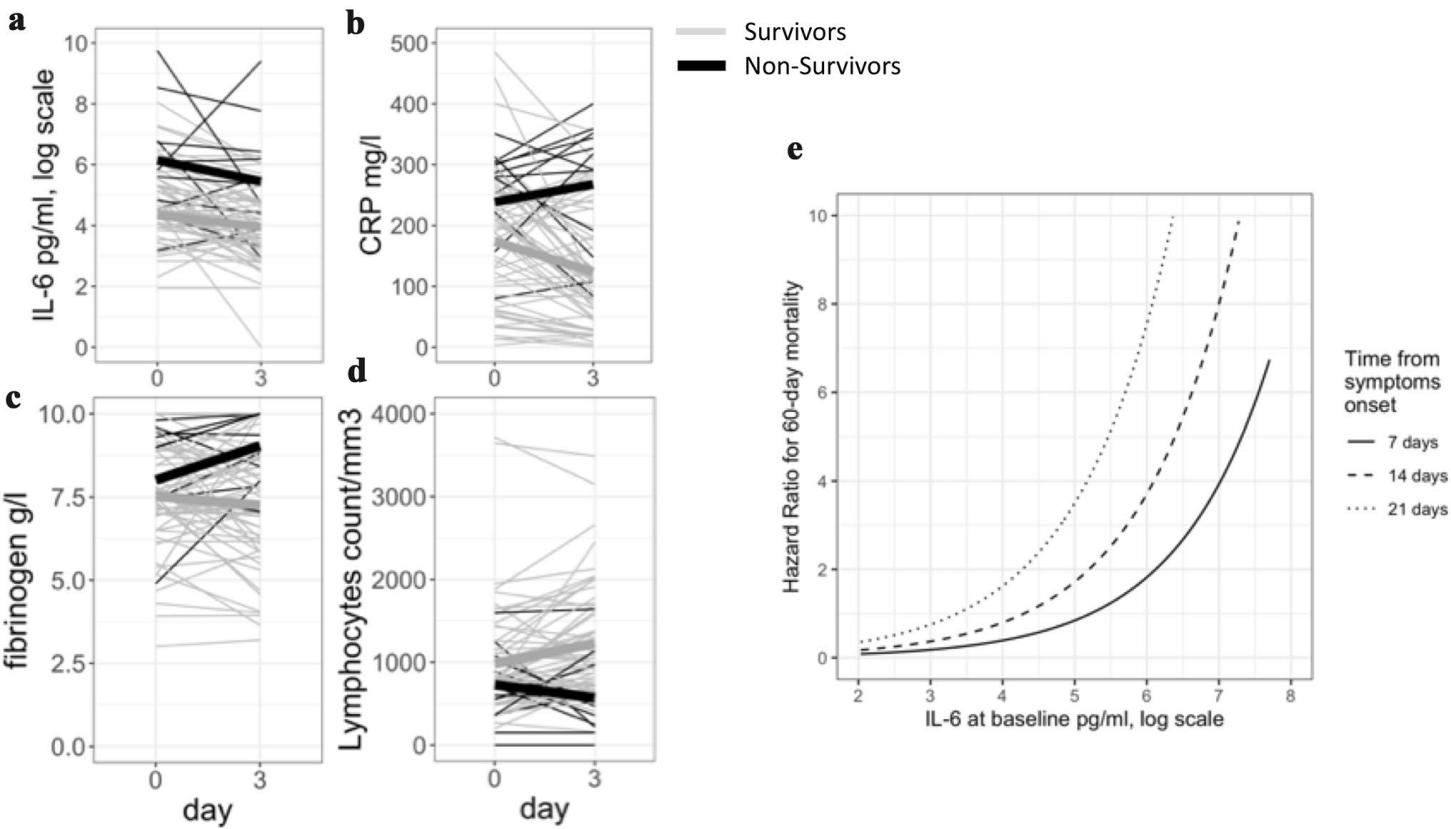
Individual trajectories of IL-6 (**a**), CRP (**b**), fibrinogen (**c**), lymphocyte count (**d**) in the blood according to the outcome. Survivors, thin grey lines; non-survivors thin black lines are represented in background; thick lines for mean values. E, adjusted effect of IL-6 level at baseline on day-60 mortality according to time from symptom onset to dosage

At day 60 after ICU admission, 19 patients (19%) had died, with a median ICU length of stay of 14 [10–19] days (Additional file 7). At admission, non-survivors had significant higher SOFA^#^ score (Table 1) and more severe hypoxemia (Additional file 2). Plasma levels of inflammatory biomarkers were measured at day 0 and day 3–4. Baseline IL-6 and CRP levels were significantly higher in non-survivors compared to survivors but fibrinogen levels and lymphocyte counts were not different between groups (Fig. 3a–d and Additional file 8). After adjustment on SOFA score and time from symptom onset to first dosage, IL-6 and CRP remained significantly associated with 60-day mortality (Table 2 and Additional file 9). IL-6 level at Day 0 was an interesting tool to identify patients at higher risk for mortality as the area under the curve was 0.80 (CI95% [0.67–0.89]) (Additional file 10), optimal IL-6 threshold was 212 pg/mL with a sensitivity of 68% (CI 95% [47–89]) and a specificity of 80% (CI95% [71–89]). The prognostic value of IL-6 was influenced by the time from symptom onset to first dosage (Fig. 3e).

**Table 2:** Analysis of predictors for mortality using multivariable Logistic Regression

	HR [95%CI]	*p*-value
IL-6 at Day 0 (log)	2.20 [1.58–3.05]	< 0.001
SOFA	1.09 [0.90–1.31]	0.40
Time between symptom onset and dosage (days)	1.09 [1.00–1.19]	0.04

*HR* Hazard Ratio, *SOFA* Sepsis-related Organ Failure Assessment, *ICU* Intensive Care Unit

IL-6 changes between Day 0 and Day 3–4 were not different according to the outcome (Fig. 3a and Table 3). However, kinetics of other studied biomarkers were interesting. CRP increased in non-survivors but decreased in survivors (Fig. 3b and Table 3). Blood lymphocyte counts remained low in non-survivors, whereas counts increased at day 3–4 in survivors (Fig. 3d and Table 3).

**Table 3:** Relationship between biomarkers variations between Day 0 and Day 3–4 and the outcome

	Survivors	Non-survivors	*p*-value for univariate comparison	Adjusted*** HR [95% CI]	*p*-value
Lymphocyte ratio*	16.4 [− 4.5, 53.3]	− 23.6 [− 54.5, 20.6]	0.02	0.9 [0.8–1.0]	0.05
Delta Lymphocyte**	− 160.0 [− 395.0, 65.0]	0 [− 40.0, 460.0]	0.01	1.2 [1.1–1.4]	0.01
CRP ratio	− 45.7 [− 62.6, 14.9]	13.9 [− 20.3, 32.1]	0.04	1.0 [0.9–1.1]	0.44
Delta CRP	39.5 [− 12.0, 114.6]	− 34.0 [− 68.7, 65.7]	0.05	0.6 [0.4–0.9]	0.01
Fibrinogen ratio	− 0.02 [− 0.88, 0.29]	0.16 [− 0.01, 0.35]	0.155	1.41 [0.77–2.57]	0,25
Delta Fibrinogen	− 0.11 [− 1.18, 0.54]	0.36 [− 0.03, 1.00]	0.05	1.40 [0.96–2.05]	0.08
IL-6 ratio	− 14.5 [− 29.3, 0.4]	− 6.6 [− 21.0, 0.2]	0.55	1.2 [0.8–1.6]	0.37
Delta IL-6	− 0.7 [− 1.6, 0]	− 0.4 [− 1.0, 0]	0.66	0.9 [0.5–1.8]	0.83

Results are given as Median [IQR] and Hazard Ratio (HR) [95%CI], *Ratio: percentage of change from Day 0 value; ** delta between Day0 and Day 3–4 values; ***Adjusted on SOFA at ICU admission and time from symptom onset to first dosage

### Impact of chronic corticosteroid treatment on inflammatory profile and outcome

In our cohort, before ICU admission, 19 patients were treated with chronic low doses of glucocorticoids with different doses and molecules. We did not observe any difference in IL-6 plasma levels between patients receiving glucocorticoids and the others (87 [37–310] vs 91 [16–252] pg/ml, *P* = 0.43). Mortality was not different between groups (3/19 vs 16/66, *P* = 0.71, Chi-squared test).

## Discussion

In this cohort study of critically ill patients with SARS-CoV-2 infection, we found that a systemic pro-inflammatory signature was associated with clinical worsening and poor outcome. Baseline high plasma levels of CRP and IL-6 were associated with both organ failure worsening and 60-day mortality, whereas baseline plasma levels of IL-1β, TNF-α did not differ with patient outcome. Kinetics of CRP and lymphocyte count were significantly different between survivors and non-survivors.

In this cohort, majority of patients were admitted to the ICU because of acute hypoxemic respiratory failure that required respiratory support. Seventy-nine percent of patients had at least one comorbidity, in agreement with recent reports in China [12], Singapore [13] and Italy [14]. Similar to other previous reports [12], hypertension was the most common comorbidity, followed by obesity and diabetes.

Besides cytokines, we measured and analysed several markers of inflammatory response in the blood. First, we measured neutrophil count that did not differ according to patient’s course or outcome. Nevertheless, we found that persistent lymphopenia was associated with in-ICU mortality. Huang et al. [4] have reported in a cohort of 41 patients with SARS-CoV-2 infections in China that blood lymphocyte count was significantly lower in critically ill patients admitted in ICU compared to patients with no-ICU care. Flow cytometry analyses showed that lymphopenia was due to decreased CD4^+^ and CD8^+^ T cells in the blood, B cell population being unchanged [5]. Lymphopenia following SARS-CoV and MERS-CoV infection injury has been previously reported [15] and is related to the activation of cell death program through both extrinsic and intrinsic pathways [16]. Experimental studies have highlighted that T lymphocytes, particularly CD4^+^ T cells, are involved in controlling and finetuning pathogenesis and outcome of coronavirus infections [17, 18]. Persistent lymphopenia may promote viral replication, explaining at least partly, the relationship between low lymphocyte blood count and poor outcome.

We measured CRP plasma levels, as a global marker of inflammation produced by the liver in response to IL-6 [19]. However, we did not find any significant correlation between IL-6 and CRP levels. Some authors have suggested that other factors modulate CRP production [20, 21]. The absence of association may also be explained by a discrepancy in the kinetics of production between both markers, IL-6 being quickly released by inflammatory cells, whereas CRP production by hepatocytes require a longer period of time [19]. Our finding suggests that CRP cannot be used as a surrogate marker for IL-6 levels in patients with SARS-CoV-2 infection. In our cohort, plasma CRP levels were higher (205 [91–263] mg/mL) than previously reported by Huang et al. (108 [28–140] mg/mL) but patient characteristics were different. Huang et al. analysed hospitalised SARS-CoV-2 + patients with and without in-ICU care, whereas we focused on selected critically ill patients with severe respiratory disease. Fibrinogen, a major coagulation factor responsible for blood clotting, is another key component of the inflammatory response [22]. Fibrinogen production by the liver is upregulated in response to cytokines such as IL-6, and released during sterile inflammation or following bacterial or viral infections. We found that high persistent plasma levels of fibrinogen were associated with in-ICU mortality. Based on this observation, we hypothesised that fibrinogen might participate in organ damage in SARS-CoV-2 infection, through its pro-inflammatory activity [22] and/or through its well-known pro-coagulant functions. High incidence of venous thrombosis and pulmonary embolism has been recently reported as a hallmark of SARS-CoV-2 infections [23].

We measured plasma levels of IL-10, a key anti-inflammatory cytokine, and 3 pro-inflammatory cytokines (IL-1β, TNF-α and IL-6). Analysis of the kinetics of plasma levels of these cytokines should help improve our understanding of SARS-CoV-2 disease and may also pave the way for effective treatment, as these cytokines (or their receptors) can be neutralised in vivo with currently available and well-validated neutralizing antibodies. In our cohort, IL-1β was not detectable in plasma, confirming a previous study in China [5]. However, given that ELISA method was not sensitive enough to detect very low plasma levels of IL-1β (Below 4 pg/mL), a pathogenic role of circulating IL-1β in SARS-CoV-2 disease could not be excluded. We did not find any relationship between TNF-α plasma levels and patient outcome, but this result does not rule out a role of this cytokine in SARS-CoV-2 induced organ failure. Indeed, plasma was analysed several days after symptom onset and TNF-α, a component of the acute-phase response, is known to be produced at earlier time points after infection. In sepsis, experimental [24] and clinical studies [25] have shown that TNF-α is produced in large amounts, but only during the first hours after bacterial exposure. Plasma levels of TNF-α are low in patients admitted in ICU later after infection, which might explain, at least in part, why TNF-α blockade using neutralizing monoclonal antibody failed to improve sepsis outcome in randomised trials [26].

In our study, we found that IL-6 plasma levels were strongly associated with disease severity. Baseline IL-6 levels in patients with SARS-CoV-2 infections were similar to those reported in patients with community acquired bacterial pneumonia [27] but lower than in patients with sepsis [28] or CRS induced by CAR T cell therapy [8]. Baseline IL-6 correlated with SOFA^#^ score and is associated with worsening organ failure. In addition, IL-6 plasma levels were significantly higher in non-survivors, supporting a pathogenic role for IL-6 in the pathophysiology of SARS-CoV-2 infection. In a retrospective multicentre study in China, elevated IL-6 plasma levels were identified as a predictive factor of mortality [29]. Such an independent association was confirmed in a large cohort of patients hospitalized in the United States [8]. Based on these results, IL-6 signalling blockade using monoclonal antibodies has been considered as an attractive therapeutic approach in SARS-CoV-2 infections that is currently under investigation in several countries using Tocilizumab [30, 31]. Our results may suggest that antibody treatment should not be based on CRP levels since CRP did not correlate with IL-6 levels. Finally, it is important to underline that possible beneficial effects of reducing inflammation should be carefully weighed up against potential deleterious impairment of anti-microbial immunity.

At baseline, IL-6 levels were predictive of poor outcome but changes at day 3–4 did not provide any additional predictive information. *A contrario*, kinetics of other inflammatory biomarkers including CRP and lymphocyte count were different between Day 0 and Day 3–4 in survivors *versus* non-survivors. However, analysis of these biomarkers at later time points was lacking in our study and should be investigated in the future.

The results of this descriptive study should be analysed with caution. We found a significant correlation between plasma inflammatory markers and COVID-19 severity. However, correlation does not imply causality. It is likely that virus replication drives inflammatory response and subsequent disease severity, the exacerbated inflammation being an inappropriate host response that requires correction. Ongoing immunotherapy trials will be helpful to confirm the pathogenic role of IL-6 in patients with SARS-CoV-2 infection.

## Conclusion

This multicentre study provides an inflammatory profiling of critically ill patients hospitalised for SARS-CoV-2 infection and shows associations between plasma inflammatory markers and both patient course and mortality. Our results may suggest a pathogenic role of IL-6 in the pathophysiology of SARS-CoV-2 infection. Future immunotherapy clinical trials will be important to confirm this hypothesis.

## Supplementary Information

**Additional file 1. **Flow chart.

**Additional file 2. **Baseline biological parameters of included ICU patients with SARS-CoV-2 infection according to 60-day outcome. Data are expressed as N (%) or median (1stIQR-3rdIQR). NS for non-significant.

**Additional file 3. **Plasma levels of IL-1β according to organ failure worsening between day 0 and day 3–4.

**Additional file 4. **Plasma levels of TNF-α and IL-10 according to time between symptom onset and cytokine dosage. Data are expressed as median with 95% CI.

**Additional file 5. **Correlation between baseline IL-6 levels and SOFA# score on patients whose time between onset symptoms and blood sampling was 9–19 days.

**Additional file 6. **No correlation between IL-6 and CRP plasma levels in ICU patients with SARS-CoV-2 infections.

**Additional file 7. **Survival curve of included patients with SARS-CoV-2 infections admitted in ICU (*N* = 101).

**Additional file 8. **Biomarker levels at baseline and at day 3–4 according to in-ICU outcome. Data are expressed as median (1st IQR–3rd IQR). a, survivors Day 0 vs Day 3–4; b non-survivors day 0 vs Day 3–4.

**Additional file 9. **Predictive value of biomarkers (Day 0) for in-ICU mortality. For each biomarker, adjustment was done on SOFA at ICU admission and time from symptom onset to measurement. HR, Hazard Ratio.

**Additional file 10. **Biomarker accuracy (at Day 0) for the prediction of mortality. AUC: Area Under Curve, LR: likelihood ratio. *Cut-off set according to Youden index method.

## Data Availability

The datasets used and/or analyzed during the current study are available from the corresponding author on reasonable request.

## Abbreviations

CRP: C Reactive Protein
ICU: Intensive care unit
IL: Interleukin
SAPS: Simplifier Acute Physiology Score
SARS: Severe Acute Respiratory Syndrome
SOFA: Sequential Organ Failure Assessment
TNF: Tumor Necrosis Factor
