# Supplementary material for: Elevated plasma IL-6 and CRP levels are associated with adverse clinical outcomes and death in critically ill SARS-CoV-2 patients: inflammatory response of SARS-CoV-2 patients
Source: Ann Intensive Care. 2021 Jan 13;11:9. doi: 10.1186/s13613-020-00798-x (PMC7804215; doi:10.1186/s13613-020-00798-x)
Supplement: Supplementary file 1 — Additional file 1. Flow chart. [file 13613_2020_798_MOESM1_ESM.pptx]

## Slide 1
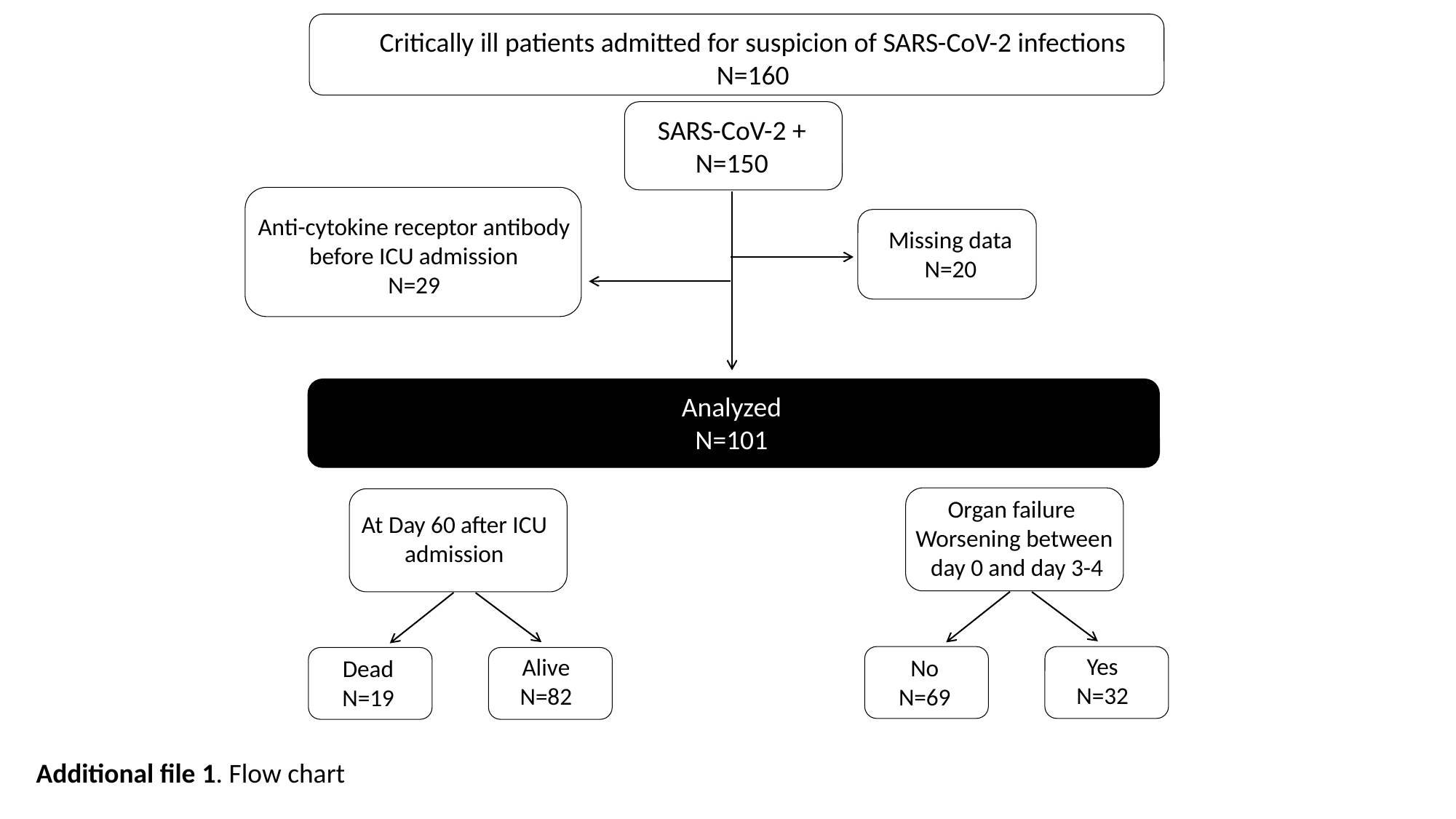

Critically ill patients admitted for suspicion of SARS-CoV-2 infections
N=160
SARS-CoV-2 +
N=150
Anti-cytokine receptor antibody before ICU admission
N=29
Missing data
N=20
Analyzed
N=101
Organ failure
Worsening between
 day 0 and day 3-4
At Day 60 after ICU admission
Yes
N=32
Alive
N=82
No
N=69
Dead
N=19
Additional file 1. Flow chart
