# Supplementary material for: Elevated plasma IL-6 and CRP levels are associated with adverse clinical outcomes and death in critically ill SARS-CoV-2 patients: inflammatory response of SARS-CoV-2 patients
Source: Ann Intensive Care. 2021 Jan 13;11:9. doi: 10.1186/s13613-020-00798-x (PMC7804215; doi:10.1186/s13613-020-00798-x)
Supplement: Supplementary file 2 — Additional file 2. Baseline biological parameters of included ICU patients with SARS-CoV-2 infection according to 60-day outcome. Data are expressed as N (%) or median (1stIQR-3rdIQR). NS for non-significant. [file 13613_2020_798_MOESM2_ESM.pptx]

## Slide 1
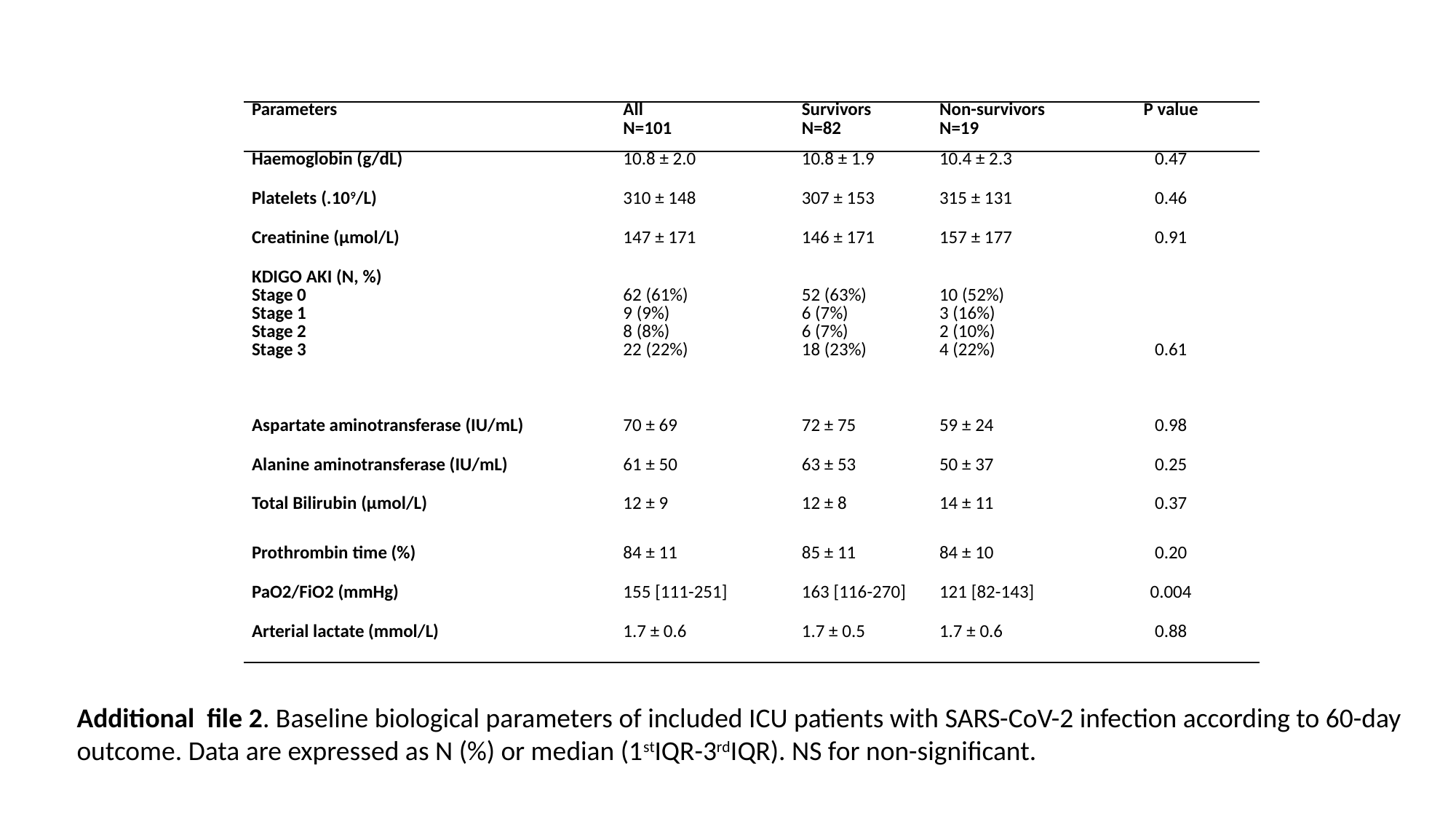

| Parameters | All N=101 | Survivors N=82 | Non-survivors N=19 | P value |
| --- | --- | --- | --- | --- |
| Haemoglobin (g/dL) | 10.8 ± 2.0 | 10.8 ± 1.9 | 10.4 ± 2.3 | 0.47 |
| Platelets (.109/L) | 310 ± 148 | 307 ± 153 | 315 ± 131 | 0.46 |
| Creatinine (µmol/L) | 147 ± 171 | 146 ± 171 | 157 ± 177 | 0.91 |
| KDIGO AKI (N, %) Stage 0 Stage 1 Stage 2 Stage 3 | 62 (61%) 9 (9%) 8 (8%) 22 (22%) | 52 (63%) 6 (7%) 6 (7%) 18 (23%) | 10 (52%) 3 (16%) 2 (10%) 4 (22%) | 0.61 |
| Aspartate aminotransferase (IU/mL) | 70 ± 69 | 72 ± 75 | 59 ± 24 | 0.98 |
| Alanine aminotransferase (IU/mL) | 61 ± 50 | 63 ± 53 | 50 ± 37 | 0.25 |
| Total Bilirubin (µmol/L) | 12 ± 9 | 12 ± 8 | 14 ± 11 | 0.37 |
| Prothrombin time (%) | 84 ± 11 | 85 ± 11 | 84 ± 10 | 0.20 |
| PaO2/FiO2 (mmHg) | 155 [111-251] | 163 [116-270] | 121 [82-143] | 0.004 |
| Arterial lactate (mmol/L) | 1.7 ± 0.6 | 1.7 ± 0.5 | 1.7 ± 0.6 | 0.88 |
Additional file 2. Baseline biological parameters of included ICU patients with SARS-CoV-2 infection according to 60-day outcome. Data are expressed as N (%) or median (1stIQR-3rdIQR). NS for non-significant.
