# Supplementary material for: Elevated plasma IL-6 and CRP levels are associated with adverse clinical outcomes and death in critically ill SARS-CoV-2 patients: inflammatory response of SARS-CoV-2 patients
Source: Ann Intensive Care. 2021 Jan 13;11:9. doi: 10.1186/s13613-020-00798-x (PMC7804215; doi:10.1186/s13613-020-00798-x)
Supplement: Supplementary file 5 — Additional file 5. Correlation between baseline IL-6 levels and SOFA# score on patients whose time between onset symptoms and blood sampling was 9–19 days. [file 13613_2020_798_MOESM5_ESM.pptx]

## Slide 1
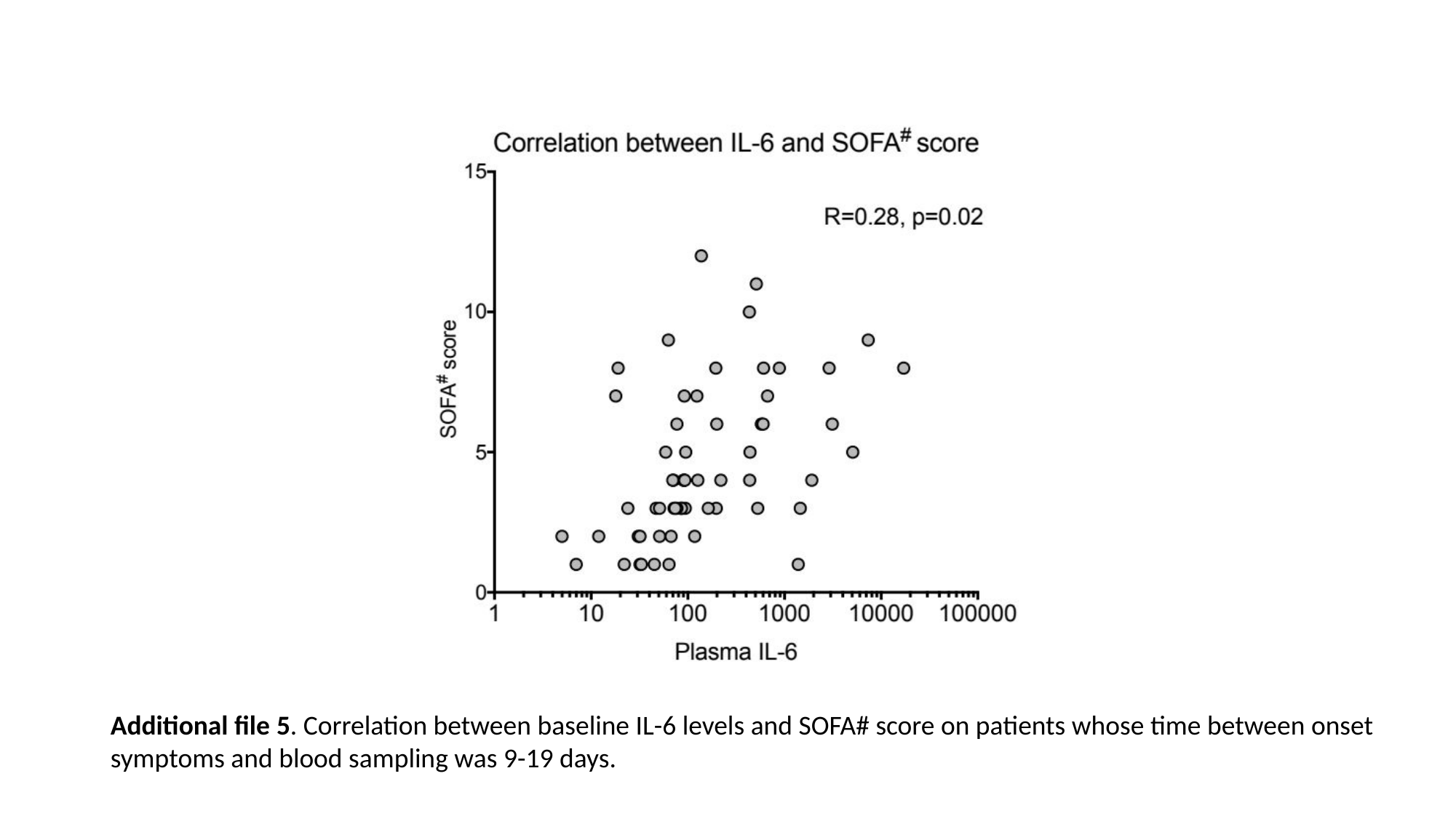

Additional file 5. Correlation between baseline IL-6 levels and SOFA# score on patients whose time between onset symptoms and blood sampling was 9-19 days.
