# Supplementary material for: Elevated plasma IL-6 and CRP levels are associated with adverse clinical outcomes and death in critically ill SARS-CoV-2 patients: inflammatory response of SARS-CoV-2 patients
Source: Ann Intensive Care. 2021 Jan 13;11:9. doi: 10.1186/s13613-020-00798-x (PMC7804215; doi:10.1186/s13613-020-00798-x)
Supplement: Supplementary file 7 — Additional file 7. Survival curve of included patients with SARS-CoV-2 infections admitted in ICU (N = 101). [file 13613_2020_798_MOESM7_ESM.pptx]

## Slide 1
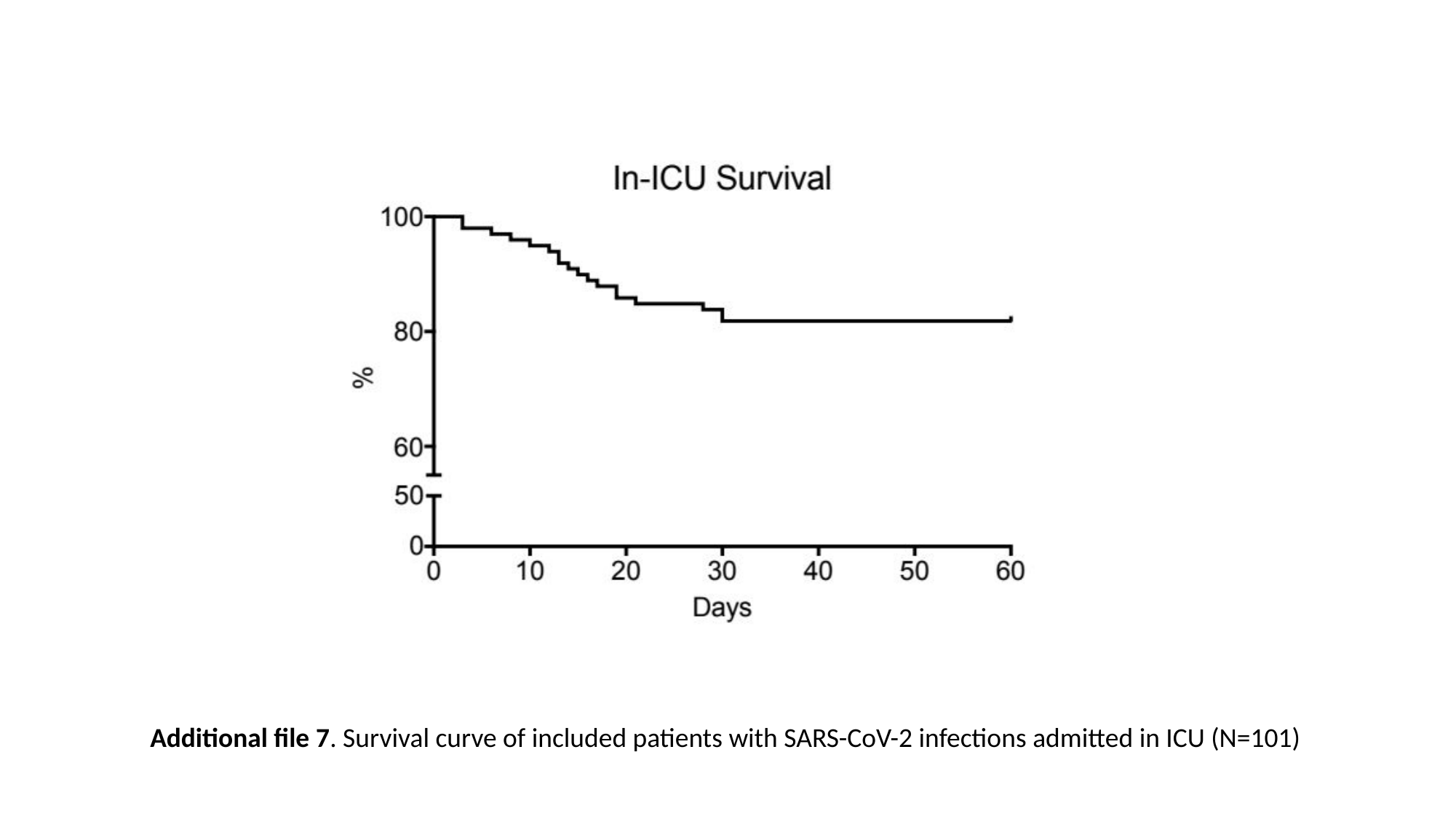

Additional file 7. Survival curve of included patients with SARS-CoV-2 infections admitted in ICU (N=101)
