# Supplementary material for: Elevated plasma IL-6 and CRP levels are associated with adverse clinical outcomes and death in critically ill SARS-CoV-2 patients: inflammatory response of SARS-CoV-2 patients
Source: Ann Intensive Care. 2021 Jan 13;11:9. doi: 10.1186/s13613-020-00798-x (PMC7804215; doi:10.1186/s13613-020-00798-x)
Supplement: Supplementary file 8 — Additional file 8. Biomarker levels at baseline and at day 3–4 according to in-ICU outcome. Data are expressed as median (1st IQR–3rd IQR). a, survivors Day 0 vs Day 3–4; b non-survivors day 0 vs Day 3–4. [file 13613_2020_798_MOESM8_ESM.pptx]

## Slide 1
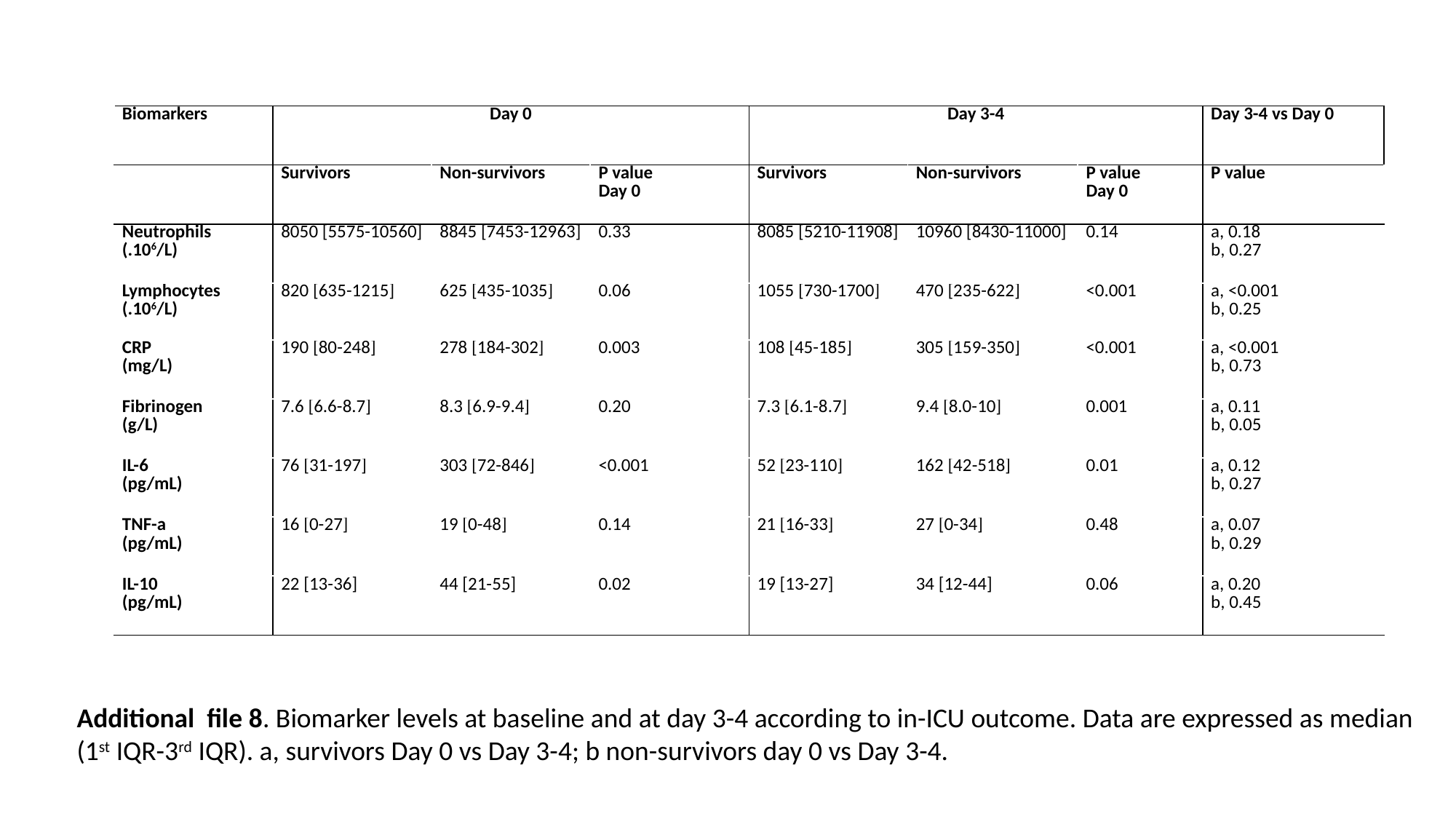

| Biomarkers | Day 0 | | | Day 3-4 | | | Day 3-4 vs Day 0 |
| --- | --- | --- | --- | --- | --- | --- | --- |
| | Survivors | Non-survivors | P value Day 0 | Survivors | Non-survivors | P value Day 0 | P value |
| Neutrophils (.106/L) | 8050 [5575-10560] | 8845 [7453-12963] | 0.33 | 8085 [5210-11908] | 10960 [8430-11000] | 0.14 | a, 0.18 b, 0.27 |
| Lymphocytes (.106/L) | 820 [635-1215] | 625 [435-1035] | 0.06 | 1055 [730-1700] | 470 [235-622] | <0.001 | a, <0.001 b, 0.25 |
| CRP (mg/L) | 190 [80-248] | 278 [184-302] | 0.003 | 108 [45-185] | 305 [159-350] | <0.001 | a, <0.001 b, 0.73 |
| Fibrinogen (g/L) | 7.6 [6.6-8.7] | 8.3 [6.9-9.4] | 0.20 | 7.3 [6.1-8.7] | 9.4 [8.0-10] | 0.001 | a, 0.11 b, 0.05 |
| IL-6 (pg/mL) | 76 [31-197] | 303 [72-846] | <0.001 | 52 [23-110] | 162 [42-518] | 0.01 | a, 0.12 b, 0.27 |
| TNF-a (pg/mL) | 16 [0-27] | 19 [0-48] | 0.14 | 21 [16-33] | 27 [0-34] | 0.48 | a, 0.07 b, 0.29 |
| IL-10 (pg/mL) | 22 [13-36] | 44 [21-55] | 0.02 | 19 [13-27] | 34 [12-44] | 0.06 | a, 0.20 b, 0.45 |
Additional file 8. Biomarker levels at baseline and at day 3-4 according to in-ICU outcome. Data are expressed as median (1st IQR-3rd IQR). a, survivors Day 0 vs Day 3-4; b non-survivors day 0 vs Day 3-4.
