# Supplementary material for: Elevated plasma IL-6 and CRP levels are associated with adverse clinical outcomes and death in critically ill SARS-CoV-2 patients: inflammatory response of SARS-CoV-2 patients
Source: Ann Intensive Care. 2021 Jan 13;11:9. doi: 10.1186/s13613-020-00798-x (PMC7804215; doi:10.1186/s13613-020-00798-x)
Supplement: Supplementary file 9 — Additional file 9. Predictive value of biomarkers (Day 0) for in-ICU mortality. For each biomarker, adjustment was done on SOFA at ICU admission and time from symptom onset to measurement. HR, Hazard Ratio. [file 13613_2020_798_MOESM9_ESM.pptx]

## Slide 1
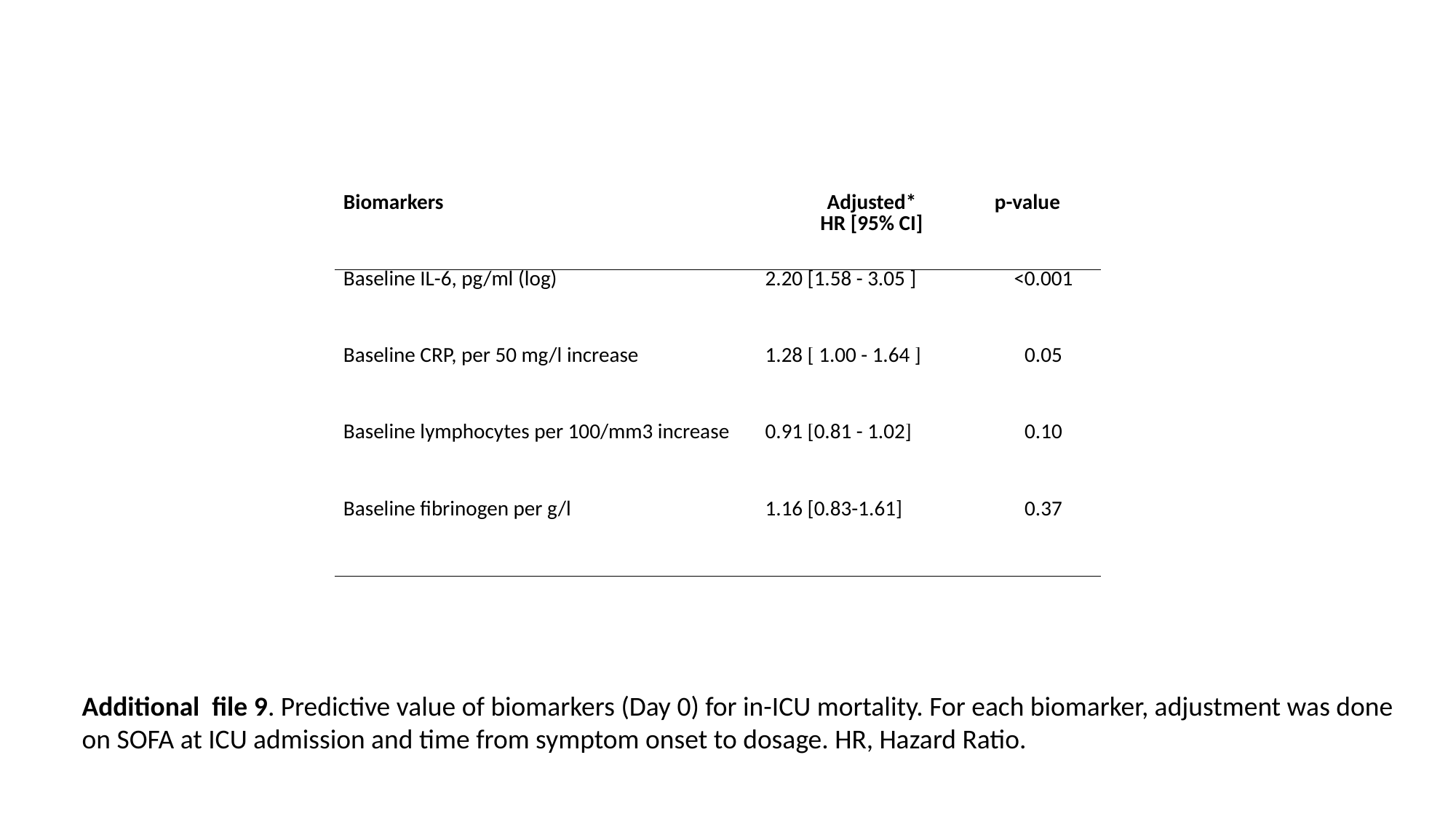

| Biomarkers | Adjusted\* HR [95% CI] | p-value |
| --- | --- | --- |
| Baseline IL-6, pg/ml (log) | 2.20 [1.58 - 3.05 ] | <0.001 |
| Baseline CRP, per 50 mg/l increase | 1.28 [ 1.00 - 1.64 ] | 0.05 |
| Baseline lymphocytes per 100/mm3 increase | 0.91 [0.81 - 1.02] | 0.10 |
| Baseline fibrinogen per g/l | 1.16 [0.83-1.61] | 0.37 |
Additional file 9. Predictive value of biomarkers (Day 0) for in-ICU mortality. For each biomarker, adjustment was done on SOFA at ICU admission and time from symptom onset to dosage. HR, Hazard Ratio.
