# Supplementary material for: Elevated plasma IL-6 and CRP levels are associated with adverse clinical outcomes and death in critically ill SARS-CoV-2 patients: inflammatory response of SARS-CoV-2 patients
Source: Ann Intensive Care. 2021 Jan 13;11:9. doi: 10.1186/s13613-020-00798-x (PMC7804215; doi:10.1186/s13613-020-00798-x)
Supplement: Supplementary file 10 — Additional file 10. Biomarker accuracy (at Day 0) for the prediction of mortality. AUC: Area Under Curve, LR: likelihood ratio. *Cut-off set according to Youden index method. [file 13613_2020_798_MOESM10_ESM.pptx]

## Slide 1
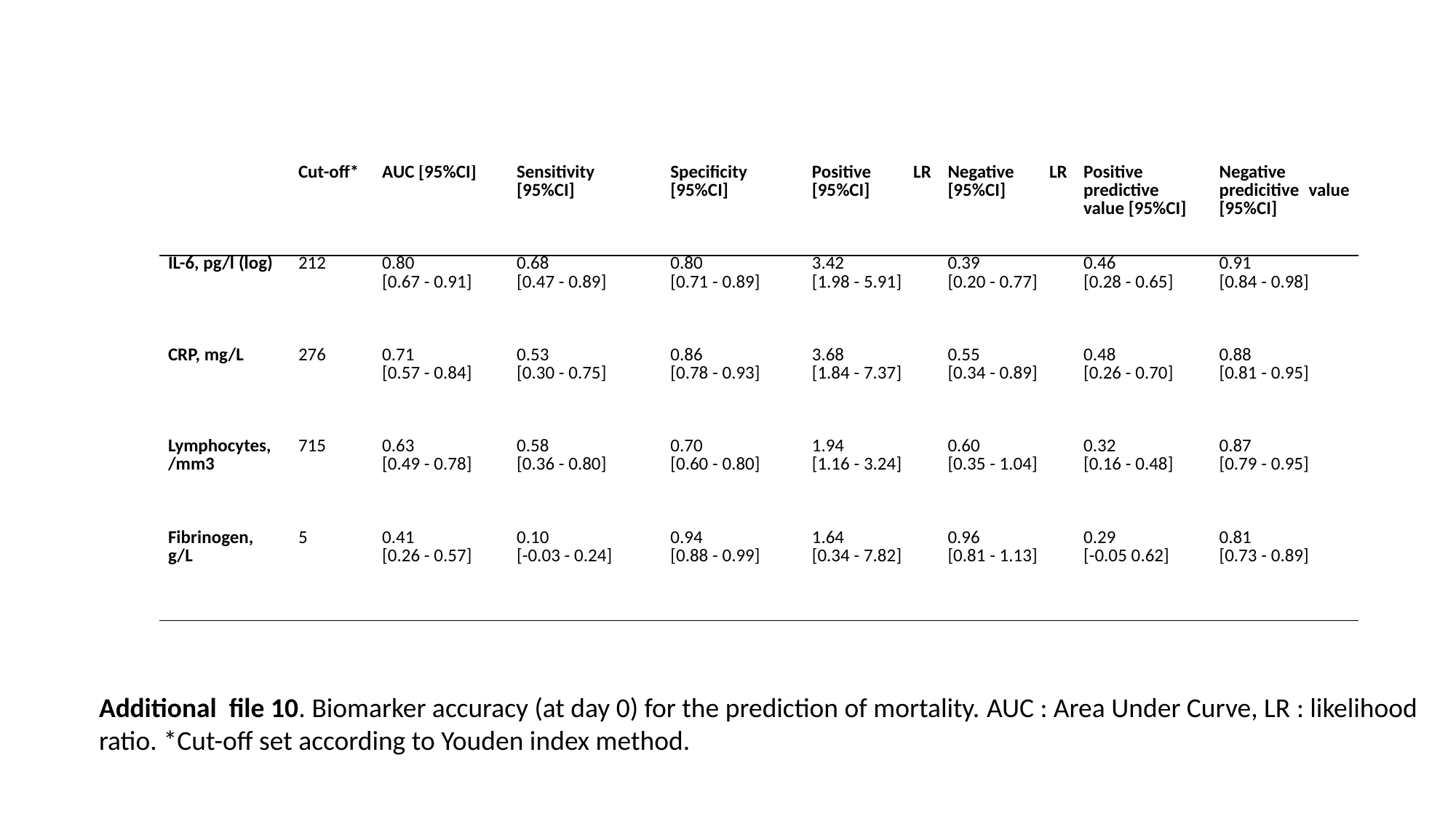

| | Cut-off\* | AUC [95%CI] | Sensitivity [95%CI] | Specificity [95%CI] | Positive LR [95%CI] | Negative LR [95%CI] | Positive predictive value [95%CI] | Negative predicitive value [95%CI] |
| --- | --- | --- | --- | --- | --- | --- | --- | --- |
| IL-6, pg/l (log) | 212 | 0.80 [0.67 - 0.91] | 0.68 [0.47 - 0.89] | 0.80 [0.71 - 0.89] | 3.42 [1.98 - 5.91] | 0.39 [0.20 - 0.77] | 0.46 [0.28 - 0.65] | 0.91 [0.84 - 0.98] |
| CRP, mg/L | 276 | 0.71 [0.57 - 0.84] | 0.53 [0.30 - 0.75] | 0.86 [0.78 - 0.93] | 3.68 [1.84 - 7.37] | 0.55 [0.34 - 0.89] | 0.48 [0.26 - 0.70] | 0.88 [0.81 - 0.95] |
| Lymphocytes, /mm3 | 715 | 0.63 [0.49 - 0.78] | 0.58 [0.36 - 0.80] | 0.70 [0.60 - 0.80] | 1.94 [1.16 - 3.24] | 0.60 [0.35 - 1.04] | 0.32 [0.16 - 0.48] | 0.87 [0.79 - 0.95] |
| Fibrinogen, g/L | 5 | 0.41 [0.26 - 0.57] | 0.10 [-0.03 - 0.24] | 0.94 [0.88 - 0.99] | 1.64 [0.34 - 7.82] | 0.96 [0.81 - 1.13] | 0.29 [-0.05 0.62] | 0.81 [0.73 - 0.89] |
Additional file 10. Biomarker accuracy (at day 0) for the prediction of mortality. AUC : Area Under Curve, LR : likelihood ratio. *Cut-off set according to Youden index method.
